# Visible Light Induced Green Transformation of Primary Amines to Imines Using a Silicate Supported Anatase Photocatalyst

**DOI:** 10.3390/molecules20021941

**Published:** 2015-01-26

**Authors:** Sifani Zavahir, Huaiyong Zhu

**Affiliations:** School of Chemistry, Physics and Mechanical Engineering, Science and Engineering Faculty, Queensland University of Technology, Brisbane QLD 4001, Australia; E-Mail: fathimasifani.zavahir@hdr.qut.edu.au

**Keywords:** benzylamine, imine, visible light, anatase, silicate

## Abstract

Catalytic oxidation of amine to imine is of intense present interest since imines are important intermediates for the synthesis of fine chemicals, pharmaceuticals, and agricultural chemicals. However, considerable efforts have been made to develop efficient methods for the oxidation of secondary amines to imines, while little attention has until recently been given to the oxidation of primary amines, presumably owing to the high reactivity of generated imines of primary amines that are easily dehydrogenated to nitriles. Herein, we report the oxidative coupling of a series of primary benzylic amines into corresponding imines with dioxygen as the benign oxidant over composite catalysts of TiO_2_ (anatase)-silicate under visible light irradiation of λ > 460 nm. Visible light response of this system is believed to be as a result of high population of defects and contacts between silicate and anatase crystals in the composite and the strong interaction between benzylic amine and the catalyst. It is found that tuning the intensity and wavelength of the light irradiation and the reaction temperature can remarkably enhance the reaction activity. Water can also act as a green medium for the reaction with an excellent selectivity. This report contributes to the use of readily synthesized, environmentally benign, TiO_2_ based composite photocatalyst and solar energy to realize the transformation of primary amines to imine compounds.

## 1. Introduction

Imines are a group of *N*-containing compounds, with a pivotal role as chemically and biologically useful intermediates in various cycloaddition, condensation and reduction reactions [1,2]. These compounds play a major role in pharmacophores, fragrances and numerous biologically active compounds [3]. For a long time, traditional condensation of amines with carbonyl compounds was regarded as the simplest way to prepare imines [4,5,6]. Highly reactive nature of aldehydes made handling difficult. This was overcome later by replacing aldehydes with alcohols and temporarily producing aldehydes *in-situ* within the reaction mixture and the subsequent reaction with an amine yield the imine compound [7,8]. Wang and co-workers have recently developed organosilicon supported TiO_2_ catalyst for this reaction at 160 °C with added base to achieve a good imine yield [9]. However, these processes yield range of by-products and greatly affect the selectivity to the desirable product. 

Amines are easily accessible compounds that can also be attractive precursors to synthesize imine by controlled oxidation. Until recently, several methods for oxidation of secondary amines to imines have been developed, while little attention has been devoted to the oxidation of primary amines. This is probably because of the generated imines, in which α-amino hydrogen is present, are generally intermediate products that are rapidly dehydrogenated to nitriles [10,11,12]. Subsequent research in the area has been dominated by the development of new catalytic processes that allow the aerobic oxidation of primary amines to imines under mild conditions. Angelici and co-workers reported aerobic oxidative homocoupling of primary amines to give imines, catalyzed by gold powder (50 µm size) and gold nanoparticles supported on alumina (5% Au/Al_2_O_3_) in toluene at 100 °C [13]. Following this study Au/C and CuCl catalysts have been employed in this reaction at 100 °C under molecular oxygen atmosphere [14,15]. It is becoming increasingly important to look for new materials that can catalyze reactions under moderate conditions (relatively low temperature and pressure). In this regard, the utilization of sunlight as an energy source to reduce the working temperature has recently attracted much attention [16,17,18]. Wang and co-workers have attained imines in excellent yields using mesoporous graphite carbon nitride photocatalyst at 80 °C [19]. In general, unavailability of structurally diverse amines has hampered the synthetic scope of oxidative coupling of benzylic amines to yield corresponding imines, yet this method is highly selective for imines.

Over the last few years, many efforts have been extended to organic redox-transformation reactions using TiO_2_ photocatalysis [20,21,22,23,24,25]. However, to date, most of the reported reactions for the synthetic transformations using TiO_2_ photocatalysts were carried out under UV irradiation and were usually associated with low selectivity [26,27]. Performing visible light induced selective transformations by photocatalysts is a challenge that has gained increasing attention. Recent discoveries demonstrated the surface modification of TiO_2_ with noble metal complexes or nanoparticles rather than bulk doping might be a better strategy in light of new visible light responsive photocatalysts that could enhance the design of efficient redox reactions under visible light irradiation. Zhao and co-workers achieved this conversion of primary amine to imine with TiO_2_ under UV light irradiation (>300 nm) [28] and later they also found it is also possible for this reaction to be initiated by visible light irradiation of λ > 420 nm [29]. According to them, amine molecules adsorbed onto TiO_2_ forms a surface complex that could absorb visible light and so initiate electron transfer and ensuing reactions. Because the reaction takes place on the TiO_2_ surface, we envisioned that ultrafine TiO_2_ powders with large specific surface areas should exhibit better catalytic activity. A feasible approach to stabilizing TiO_2_ nanocrystals is to disperse them in an inorganic medium, such as layered clays creating porous composite structures, while ensuring that most of the surface of TiO_2_ crystals is accessible to various molecules [30]. Nevertheless, the structure of the composite solids has a profound impact on their catalytic performance [31]. The mesoporous composites of anatase nanocrystals and silicate are the catalyst materials of the optimal structure for high photocatalytic activity. Synthetic layered clay, laponite, can be used in the synthesis of the composite as silicate source [30].

Here in we report TiO_2_ nanocrystal-silicate composite, prepared using laponite, as feasible photocatalyst for the selective oxidation of benzylamine to *N*-benzylidene benzylamine with excellent conversion and selectivity under the irradiation of visible light (λ > 460 nm). It is found that in the TiO_2_-silicate composite catalyst TiO_2_ is in anatase phase. Reference reactions with anatase show that under identical conditions TiO_2_-silicate composite catalyst exhibited a much more superior catalytic activity to pure TiO_2_ (anatase) powder. Nitrogen adsorption data confirms the large surface area of the composite catalyst. Furthermore, we also found that water can be used as the solvent. This catalyst could be employed for heterocoupling of two structurally diverse amines in the synthesis of imines as well as homocoupling of benzylic amines to imines, and the catalyst can be recycled up to five rounds without any significant loss of activity. 

## 2. Results and Discussion

The aerobic photocatalytic oxidation of benzylamine to *N*-benzylidene benzylamine was chosen as the model reaction to optimize the reaction system. Reactions are carried out using 500 W halogen lamps where the light emitted is in 400–800 nm range. According to the data given in Table 1 it is apparent that TiO_2_-silicate (abbreviated as TiO_2_-S hereafter) is the most photocatalytically active photocatalyst towards this transformation. Catalyst samples were also prepared by loading Au and Pd nanoparticles (NPs) (3% by weight) and another sample with AuPd alloy NPs (1.5% weight of each metal) loaded on to TiO_2_-S composite material (Characterization is provided in SI). We observed a lower imine product yield of 60% with Au@TiO_2_-S, compared to 82% by TiO_2_-S, despite the enhanced light absorption by Au NPs in the visible region due to localized surface plasmon resonance (LSPR) effect which is characterized by an intense band around 520 nm (Figure S1, Supplementary Information) [16,17,18]. This observation also serves as an example to support the fact, light absorption by a material is not the sole governing factor deciding catalysts ability to drive a particular chemical reaction under light irradiation. It appears that the reaction takes place on the surface of anatase, the loaded Au NPs lower the exposed surface area of TiO_2_, the catalytically active sites of this system, lowering the accessibility to the reactants. Pd@TiO_2_-S catalyst had similar activity to that of TiO_2_-S, whereas AuPd@TiO_2_-S was slightly sluggish. Results further indicate the unique potential of TiO_2_ based materials towards oxidation reactions and importance of evaluation of surface modifications of TiO_2_ for activity improvements. In control experiments, the reaction did not proceed without a photocatalyst or in the dark. 

**Table 1:** Photocatalytic oxidation of benzylamine to *N*-benzylidene benzylamine over different catalyst materials and solvents. ^a^ 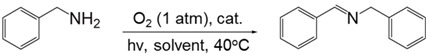

Entry	Catalyst	Solvent	Conv. (%) ^b^	Sel. (%) ^b^	Yield (%)
1	TiO_2_-S	Acetonitrile	88	92	81
2	TiO_2_-S	DMSO	18	100	18
3	TiO_2_-S	THF	94	73	69
4	TiO_2_-S	Toluene	74	97	72
5	Au@TiO_2_-S	Acetonitrile	65	93	60
6	AuPd@TiO_2_-S	Acetonitrile	88	90	79
7	Pd@TiO_2_-S	Acetonitrile	89	96	85
8	Laponite	Acetonitrile	0	--	0
9	TiO_2_(anatase)	Acetonitrile	51	100	51
10	H-titanate	Acetonitrile	73	97	71

^a^ Reaction conditions: 50 mg catalyst, 0.5 mmol benzylamine, 5 mL solvent, 500 W halogen lamp (cut off wavelength below 400 nm) intensity 0.36 W/cm^2^, 1 atm O_2_, 24 h. ^b^ Determined by GC analysis. DMSO = dimethyl sulfoxide, THF = tetrahydrofuran.

**Figure 1:**
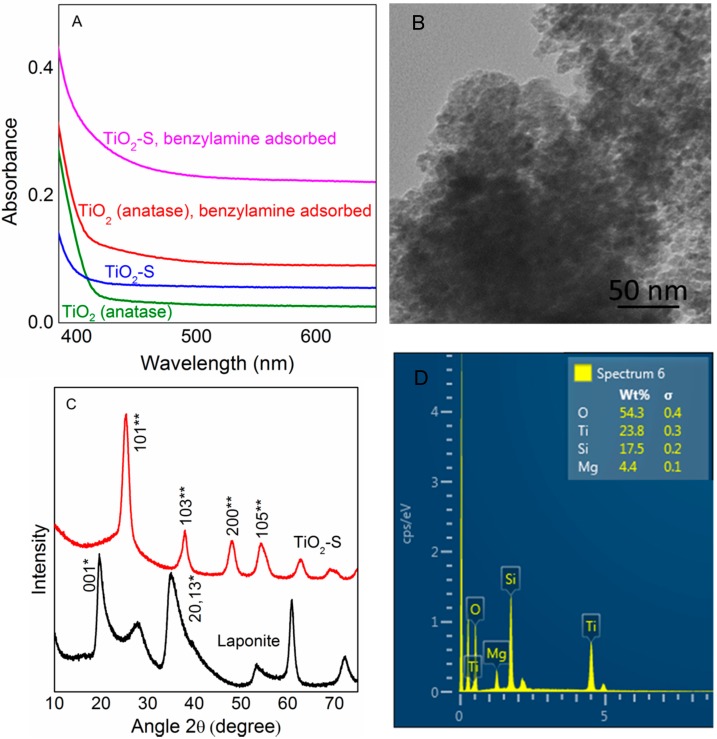
(**A**) UV-Visible diffuse reflectance spectra; (**B**) transmission electron microscopy (TEM) image; (**C**) X-ray diffraction (XRD) peak patterns indexed for *—silicate phase and **—anatase phase; (**D**) energy dispersive X-ray (EDX) spectra of TiO_2_-S composite catalyst.

As can be seen in Table 1, activity of TiO_2_-S is superior to that of an equivalent amount of TiO_2_ (anatase) as the photocatalyst material. In order to understand this change in behavior we closely studied the light absorption abilities of both TiO_2_ (anatase) and TiO_2_-S, in the presence and absence of benzylamine. UV-Visible diffuse reflectance spectra of benzylamine adsorbed TiO_2_ (anatase) and TiO_2_-S shows increased absorbance compared to solitary TiO_2_ (anatase) and TiO_2_-S, particularly in the visible region. This observation agrees well with previous reports, where electron rich molecules like amines make a charge transfer complex with TiO_2_ and respond to visible light illumination [29]. It is also notable, the absorption of benzylamine adsorbed on TiO_2_-S is significantly high compared to benzylamine adsorbed on TiO_2_ (anatase) as shown in the Figure 1A. Even though TiO_2_ is present in anatase phase in both TiO_2_ and TiO_2_-S photocatalysts used in the current study, the distribution of anatase particles is different in TiO_2_-S. During TiO_2_-S preparation, layered clay structure of precursor material laponite clay is lost as a result of the acidic titanium sol solution reacting with hydroxyl groups in the clay layers that are bound to magnesium ions within the layer [31]. Most of the magnesium in the clay was leached out in this way. Composition of the catalyst estimated by energy dispersive X-ray (EDX) confirms high weight percentage of silicate in the composite catalyst despite the leaching of Mg units. During preparation Si:Mg ratio (by weight) decreased from 1:0.58 to 1:0.25, this together with TEM image is a clear indication that ordered layer structure is damaged. Thus, TiO_2_ in this TiO_2_-S composite catalyst exists as discrete anatase crystals on fragmentized pieces of silicate. Correspondingly this composite structure restrains agglomeration of anatase particles leading to high exposed surface area of TiO_2_. Brunauer-Emmett-Teller (BET) surface area of initial laponite clay changed from 330.6 m^2^g^−1^ to 518.3 m^2^g^−1^ in the final TiO_2_-S catalyst material. The composite has porosity of about 0.4 cm^3^/g and a mean pore size of 5 nm. Finally, in the obtained composite catalyst silica particles and anatase crystals exist as inter-dispersed phases in nanometer scale with a highly porous structure as can be seen in Figure 1B. X-ray diffraction (XRD) pattern of the catalyst only exhibit peaks responsible for the anatase phase of TiO_2_ with no peaks related to silicate units or laponite clay, this indicates silica is present in the amorphous phase, and anatase particles of mean crystal size 4.22 nm (estimated by Debye-Scherrer equation using the broadening of the highly intense (101) XRD peak at 2θ = 25.3°) have homogeneously crystallized over amorphous silica moiety. This TiO_2_-S structure obtained in the present study, offers high thermal and chemical stability, also provides ample opportunity for the reactant molecules to interact with energetic charge carriers. Smaller anatase particles reduce the possibility of charge recombination, since charge carriers are generated at the close proximity of surface and efficiently captured by benzylamine and oxygen molecules on the surface.

**Figure 2:**
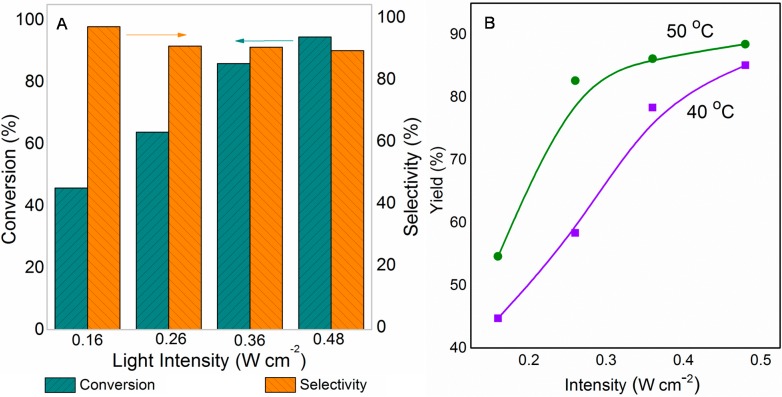
The effect of (**A**) light intensity and (**B**) temperature influence of the irradiation on the oxidative coupling of benzylamine.

To further investigate the contribution from light in this reaction, we conducted a series of reactions at variable intensities (Figure 2A). The conversion rate of benzylamine on TiO_2_-S catalyst increased gradually as the intensity increased, with the other reaction conditions unchanged (Experimental section). Selectivity to the product imine had a little influence on the intensity; however, overall imine yield (conversion rate x selectivity) increased with the intensity. Such a tendency reveals a strong dependence on the intensity for the light induced oxidative coupling of benzylamine, because in general light incident with a higher intensity is able to generate more energetic charge carriers (holes and electrons). Such conditions favor stronger interaction between benzylamine and the catalyst, and positively influence the reaction.

Impact of the temperature on the yield of the reaction was studied by conducting the experiments at two different temperatures slightly above room temperature (40 °C and 50 °C). As shown in Figure 2B, observed yield was always high for the reaction under the study, oxidative coupling of benzylamine to imine at higher temperature for a given constant intensity within 40 to 50 °C temperature range. For instance, at 40 °C and 0.26 W·cm^−2^ intensity, 58% of benzylamine was converted to imine product whereas at 50 °C it was 83%. At high temperature however, the enhancement in the yield by increasing the intensity lessens since the selectivity to the imine product starts to decline, though reaction proceeds at a higher conversion rate. As the intensity was increased, the difference between the yields at 40 and 50 °C finally decreased, even though higher conversion rate was observed for 50 °C compared to that at 40 °C in all cases.

**Figure 3:**
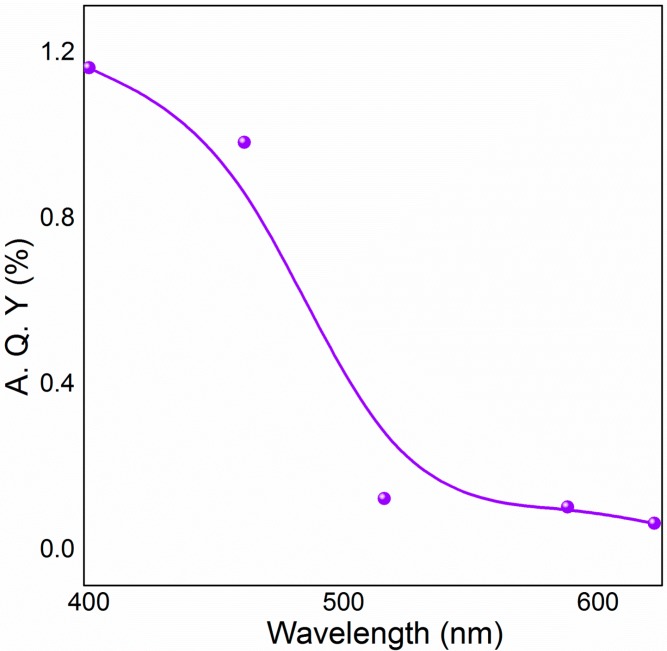
The effect of wavelength of the irradiation on the oxidative coupling of benzylamine.

The dependence of yield on the irradiation wavelength was studied using five monochromatic light emitting diodes (LEDs) and it shows that higher photocatalytic yields are achieved under irradiation of short wavelengths (<460 nm). Anatase phase of TiO_2_ exhibits a band gap of 3.2 eV (387.5 nm) where as in this case TiO_2_-S is highly active up to 460 nm. Figure 3 demonstrates the apparent quantum yield (A.Q.Y) dependence on the incident wavelength; A.Q.Y. is a measure of imine yield per photon of energy absorbed per unit time. This finding indicates that composite TiO_2_-S catalyst structure has a broad light response below 460 nm in the visible region due to the collective effects of benzylamine adsorbed TiO_2_ (anatase) charge transfer surface complex and high population of defects in the composite photocatalyst structure. This reveals that TiO_2_-S catalyst can function at a lower cut-off edge (460 nm) compared to 420 nm cut-off for solitary TiO_2_ (anatase) system reported by Zhao and co-workers [29]. It is noteworthy that, in the composite structure of the catalyst there are contacts between silicate and anatase crystals. At these sites, the anatase surface is similar to the silica doped anatase surface that exhibits light absorption and visible light photocatalytic activity [32]. 

According to the results summarized in Table 1, the solvent has important impact on the catalytic activity. Acetonitrile serves as the best solvent for this reaction while the poorest performance was observed in DSMO. Water is a viable solvent for organic reactions, and it is interesting to study the impact of water as the reaction medium. For some organic reactions, water exhibits special reactivity or selectivity due to its unique physical properties. In the present study, benzylamine oxidation reaction proceeded in water at a moderate conversion rate of 45.6% under the illumination of 500 W halogen lamp (400–800 nm) for 24 h, but with an excellent selectivity where the sole product being *N*-benzylidene benzylamine with an overall yield of 46%. Doubling the amount of catalyst from 50 mg to 100 mg of TiO_2_-S enhanced the reaction yield to 62% without compromising the selectivity. 

Motivated by this result, we expanded the scope of the substrates for the oxidation of amines to imines. Table 2 summarizes the photocatalytic oxidation of the benzylic amines to corresponding imines with their conversion rate and selectivity. Oxidation of primary benzylic amines substituted with an electron donating group (Table 2, entry 2–3) proceeded efficiently under visible light irradiation with good to high conversion rates and high selectivity for the imine product. Substituent group influences the conversion rate of the reaction than the selectivity to the imine product. The relatively low conversion rate for the oxidative coupling of 4-chlorobenzylamine (Table 2, entry 4) into its corresponding imine might be caused by inductive effects of C-Cl σ-bond polarity. No change was observed when aniline was subjected to the reaction, this is consistent with our hypothesis that the presence of a –H, bonded to the α-carbon is important for this transformation to take place. Furthermore, non-aromatic cyclic amines (Table 2, entry 7–8) did not yield the desired imine product. Control experiments carried out using cyclohexylamine (consist of a single α-hydrogen) produced the corresponding oxime (cyclohexanone oxime) instead of the imine. Benzaldehyde oxime was one of the products observed during the time course of the reaction of benzylamine.

**Table 2:** Aerobic oxidation of primary benzylic amines photocatalyzed by TiO_2_-S under visible light irradiation. ^a^ 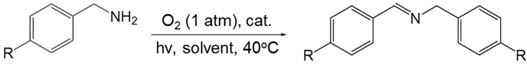

Entry	Substrate	Product	Con. (%) ^b^	Select. (%) ^b^	Yield (%)
1	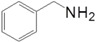	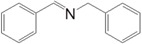	88	92	81
2	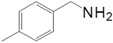	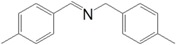	96	92	88
3	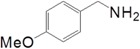	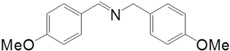	100	96	96
4	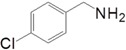	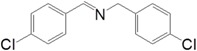	57	100	57
			88 ^c^	>99 ^c^	87 ^c^
5	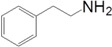	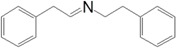	91	35	32
7	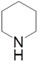	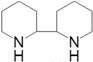	51	20	10
8	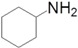	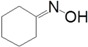	74	48	36

^a^ Reaction condition: 50 mg catalyst, 0.5 mmol amine substrate, 5 mL acetonitrile, 500 W halogen lamp (cut-off wavelength below 400 nm) intensity 0.36 W/cm^2^, 1 atm O_2_, 24 h. ^b^ Determined by GC analysis. ^c^ Reaction time 36 h.

**Scheme 1:**
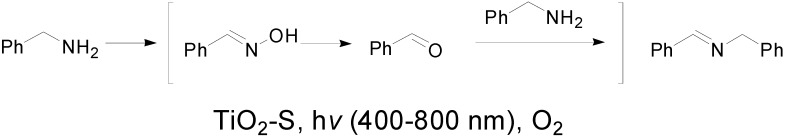
Tentative reaction pathway.

This reaction goes through the widely known intermediate benzaldehyde and a tentative mechanistic pathway is given in Scheme 1 based on the products observed. The photocatalysts contribution is mainly in the step of benzylamine oxidation to benzaldehyde, whereas the condensation of benzaldehyde with a benzylamine molecule leading to the imine product is faster. In the oxidation step, TiO_2_-benzylamine surface complex absorb visible light (400–800 nm) and excite electrons. These excited electrons are then captured by oxygen molecules adsorbed on TiO_2_ surface, then in the proceeding steps oxygen interacts with benzylamine and the substrate molecules lose the H bonded to the α-carbon atom, and oxidized to aldehyde. Thus, it is rational that under visible light irradiation, the oxygen molecules adsorbed on the catalyst capture the light excited electrons, and react with the H at the α-carbon. Role of oxygen is further confirmed, when the reaction was carried out in the air atmosphere benzylamine exhibit a relatively lower observed conversion rate of 51% and a selectivity of 96%, yielding 49% of imine after 24 h. This mechanism agrees well with the observed product selectivity results. At higher conversions of benzylamine, a decrease in the selectivity for the imine occurs and benzaldehyde appears in the products. This is due to the fact that, increased consumption of benzylamine in the solution could not ensure the complete condensation of aldehyde and the amine.

Ability of TiO_2_-S photocatalyst to catalyze the oxidative cross-coupling of two benzylic amines with different substituent groups to yield a heterocoupled imine product was also studied using benzylamine, 4-methylbenzylamine and 4-methoxybenzylamine (two at a given reaction). Results demonstrated a poor selectivity since all four possible imines were observed in relatively similar yields after 24 h. Oxidative coupling of benzylamine with 4-methylbenzylamine had 95% of imine product yield. Self-coupling products of benzylamine (28%) and 4-methylbenzylamine (22%) were observed together with the two heterocoupled imines (50%), the product distribution of heterocoupling of benzylamine with 4-methylbenzylamine is as desired (~1:1:1:1) since difference in the nucleophilicities of “H” and methyl group is not significant. In order to evaluate the product distribution over the time span of this heterocoupled imine synthesis, we chose benzylamine and 4-methoxybenzylamine as the two benzylic amine substrates and the reaction profile is given in the Table 3, this reveals both the precursor imines produce the corresponding aldehydes as per the oxygenation step shown in Scheme 1, and then reacts with a free amine molecule to yield the final imine. Aldehyde of more electro deficient nucleus reacts faster with the more electron rich amine (P3) at early stages of the reaction and then with either amine as the reaction is progressing. Rate of aldehyde formation is slower in electron rich benzene nucleus, benzylamine in this system and it acts as the nucleophile (amine half), while 4 methoxybezylamine is easier to oxidize and preferentially be the aldehyde half. In the product distribution more P3 and P4 are observed during the whole cause of the reaction indicating high formation and reactivity of 4-methoxybenzaldehyde. Dual amine systems of benzylamine/aniline and 4-methoxybenzylamine/aniline yield only the self-coupled imines of benzylamine (92%) and 4-methoxybenzylamine (96%) respectively. Amount of aniline introduced in the reaction system remained unchanged even after the reaction, portraying its inert role in this photocatalyzed oxidative coupling reaction, anilne with a –NH_2_ unit in its structure failed to participate in this heterocoupling reactions at least as the amine half. 

**Figure 4:**
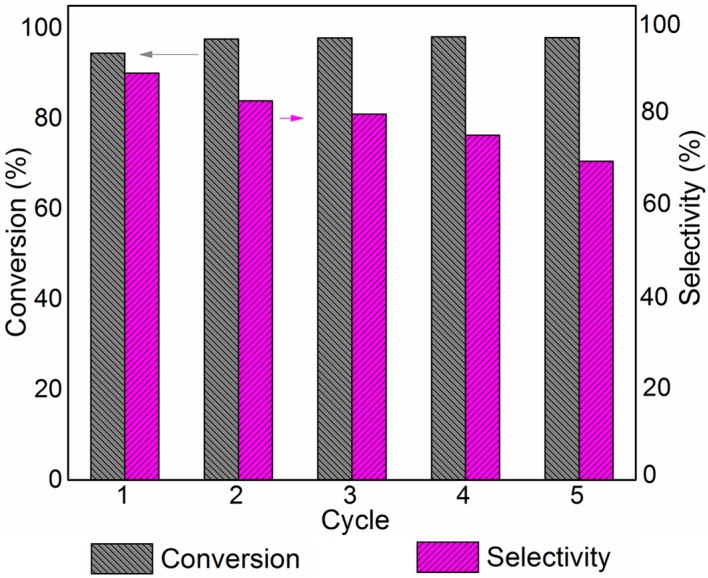
Reusability data of TiO_2_-S catalyst for the oxidative coupling of benzylamine.

Reusability of the catalyst is an important parameter in heterogeneous catalysis. The composite TiO_2_-S photocatalyst studied in this system can be recovered readily from aqueous or organic solutions by simple filtration or sedimentation. The anatase nanocrystals in these composite samples are linked to silicate pieces such that grains in the µm scale are formed. Operational life of this catalyst examined over five consecutive rounds (Figure 4) revealed no apparent activity loss after five rounds. This further confirms the thermal and chemical stability of the catalyst. However selectivity towards the imine product was gradually decreased during each cycle lowering the overall product yield. 

**Table 3:** Time conversion plot for oxidative coupling of benzylamine with 4-methoxybenzylamine ^a^.

Entry	Time (h)	Conversion (%)		Selectivity (%)			
		Benzylamine	4-Methoxybenzylamine	P1	P2	P3	P4
1	2	13	13	0	0	100	0
2	4	41	57	12	12	40	36
3	8	84	90	15	16	38	32
4	17	96	97	19	16	40	26
5	20	96	97	21	16	40	24

^a^ Reaction Conditions: 25 mg catalyst, 0.25 mmol amine substrates, 2 mL acetonitrile, 500 W halogen lamp (cut-off wavelength below 400 nm) intensity 0.36 W/cm^2^, 1 atm O_2_.
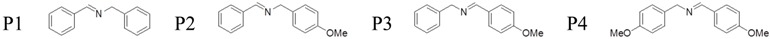

## 3. Experimental Section

### 3.1. General Information and Materials

The laponite clay was supplied from Fernz specialty chemicals Australia, all other chemicals were purchased from Sigma Aldrich (Castle Hill NSW, Australia) and used as received without further purification. Water used in all experiments was milli-Q water passing through an ultra-purification system.

### 3.2. General Procedure for the Synthesis of TiO_2_-S Composite

TiO_2_ precursor was prepared by hydrolyzing Ti(OCH_3_)_4_ in HCl for 3 h following a slightly modified method proposed by J. Sterte [30,31,33].

Initially 1.0 g of laponite was slowly dispersed in 50 mL of deionized water and kept stirring until it was transparent. Then 4.0 g of polyethylene glycol (FW 585) surfactant and the metal precursor solution was added drop wise with continuous stirring. Mixture was then transferred to teflon covered autoclaves and heated at 100 °C for 2 days. The solid was then recovered from centrifugation, followed by washing with water until no more chloride ions left (confirmed by a test with AgNO_3_). Product was then dried in air and finally calcined at 500 °C for 20 h with the step being 2 °C·min^−1^.

### 3.3. Characterization of TiO_2_-S Composite

The diffuse reflectance UV/Vis (DR-UV/Vis) spectra were recorded on a Cary 5000 UV/Vis-NIR Spectrophotometer (Agilent, Santa Clara, CA, USA). X-ray diffraction (XRD) patterns of the samples were recorded on a Philips PANalytical X’Pert PRO diffractometer (PANalytical, Sydney, Australia) using CuKα radiation (l = 1.5418 Å) at 40 kV and 40 mA. Transmission electron microscopy (TEM) images were taken with a Philips CM200 Transmission electron microscope (Philips, Eindhoven, The Netherlands) employing an accelerating voltage of 200 kV. The specimens were fine powders deposited onto a copper micro grid coated with a holey carbon film. Nitrogen physisorption isotherms were measured on the Tristar II 3020 (Micromeritics, Norcross, GA, USA). Prior to the analysis, sample was degassed at 110 °C overnight under high vacuum. The specific surface area was calculated by the Brunauer-Emmett-Teller (BET) method from the data in a P/P° range between 0.05 and 0.2. The compositional data was determined by energy-dispersive X-ray spectroscopy (EDS) (EDAX, Mahwah, NJ, USA ) attached to an FEI Quanta 200 scanning electron microscope (SEM, Quanta, OR, USA).

### 3.4. General Procedure for the Photocatalytic Reactions

Benzylic amine compound 0.5 mmol, 5 mL of solvent were measured to a clean dry reactor tube. 50 mg of the catalyst was added and finally the reactor was purged with oxygen gas. These reactors were kept magnetically stirring in front of a 500 W halogen lamp (except for dark and wavelength experiments) for 24 h at 40 °C. At the end of the reaction 1 mL samples were collected in to small glass vials after filtering out the solid catalyst using 0.2 µm milli pore filter. We tested for the products using a gas chromatograph (GC, Agilent, Santa Clara, CA, USA) equipped with a DB 5 column. For wavelength experiments, 5 monochromatic light emitting diodes (LEDs) of 390–410 nm, 460–462 nm, 515–517 nm, 587.5–560 nm or 620–625 nm was used.

## 4. Conclusions

We have successfully applied TiO_2_-S composite photocatalyst in the oxidative coupling of benzylamine to imine under visible light irradiation. The numerous contacts between the anatase crystals and silicate and high population of defects in the composite photocatalyst are the possible reasons behind the enhanced visible light activity. The formation of imines proceed via an oxidation pathway: under visible light irradiation, the oxygen molecules adsorbed on the catalyst capture the light excited electrons, and react with the H bonded to the α-carbon of the substrate molecules, which is oxidized to aldehyde. The condensation of the aldehyde with amine yields the product imine. This photocatalyst has a very high activity in the region λ > 460 nm. This range is much broader compared to previously reported results for anatase materials (λ > 420 nm). Intensity, wavelength and reaction temperature can be tuned to optimize the reaction rate of TiO_2_-S catalyzed oxidative coupling of benzylic amines. Water can be used as a solvent giving moderate conversion rate but sole product. These findings encourage us to further study the surface modified titania based materials for selective organic synthesis. 

## Supplementary Materials

Supplementary materials can be accessed at: http://www.mdpi.com/1420-3049/20/02/1941/s1.
