# Supplementary material for: Visible Light Induced Green Transformation of Primary Amines to Imines Using a Silicate Supported Anatase Photocatalyst
Source: Molecules. 2015 Jan 26;20(2):1941–54. doi: 10.3390/molecules20021941 (PMC6272701; doi:10.3390/molecules20021941)
Supplement: Supplementary file 1 [file molecules-20-01941-s001.pdf]

Supporting Information

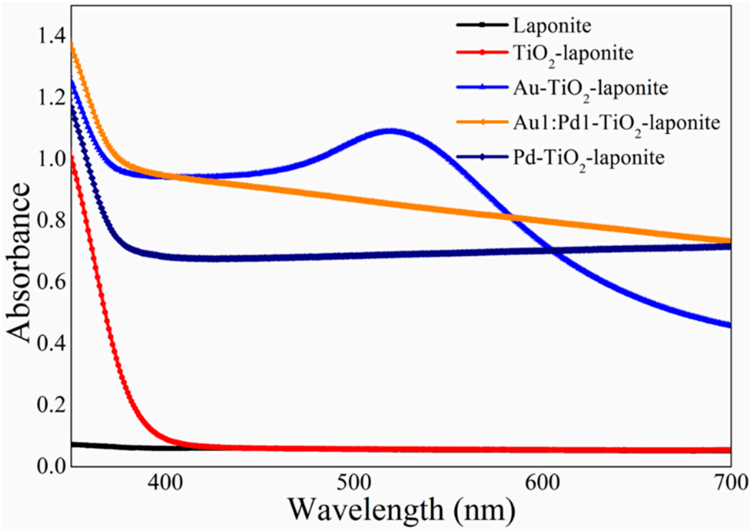

Figure S1. UV-Visible diffuse reflectance spectra of the metal loaded photocatalyst samples.

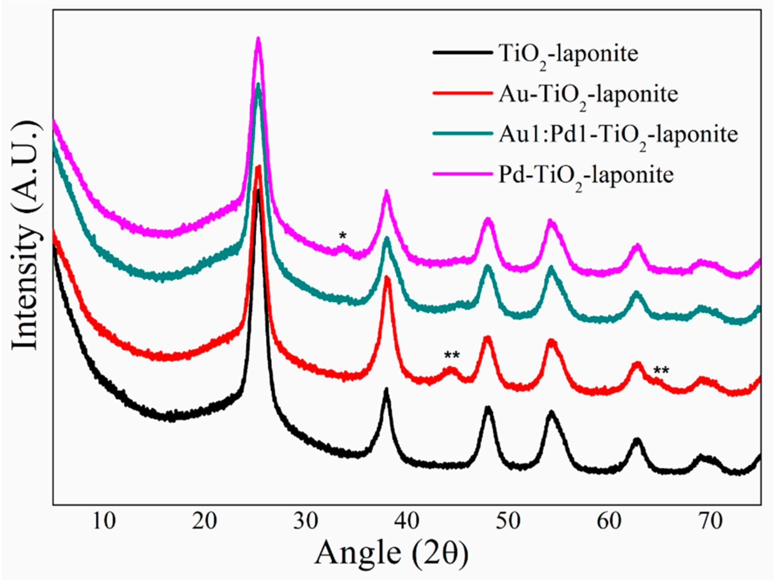

Figure S2. XRD peak patterns of the metal loaded photocatalyst samples. *—peak related to Pd (2θ = 34°), **—peaks related to Au [002], [022].

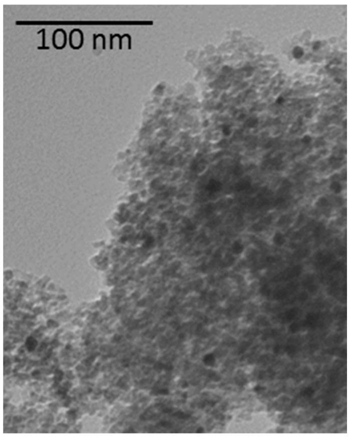

Figure S3. TEM image of 3% Au@ TiO_2-S.
